# Association of Anti–Tumor Necrosis Factor Therapy With Mortality Among Veterans With Inflammatory Bowel Disease

**DOI:** 10.1001/jamanetworkopen.2021.0313

**Published:** 2021-03-01

**Authors:** Shirley Cohen-Mekelburg, Beth I. Wallace, Tony Van, Wyndy L. Wiitala, Shail M. Govani, Jennifer Burns, Rachel Lipson, Huifeng Yun, Jason Hou, James D. Lewis, Jason A. Dominitz, Akbar K. Waljee

**Affiliations:** 1 Department of Internal Medicine, University of Michigan, Ann Arbor; 2 Veterans Affairs Center for Clinical Management Research, Veterans Affairs Ann Arbor Healthcare System, Ann Arbor, Michigan; 3 Institute for Healthcare Policy and Innovation, University of Michigan, Ann Arbor; 4 Department of Medicine, Division of Gastroenterology, South Texas Veterans Healthcare System, San Antonio; 5 Department of Medicine, Division of Gastroenterology, UT Health San Antonio, San Antonio, Texas; 6 Department of Epidemiology, School of Public Health, University of Alabama at Birmingham; 7 Center for Innovations in Quality, Effectiveness, and Safety, Michael E DeBakey Veterans Affairs Medical Center, Houston, Texas; 8 Section of Gastroenterology and Hepatology, Department of Medicine, Baylor College of Medicine, Houston, Texas; 9 Center for Clinical Epidemiology and Biostatistics, University of Pennsylvania, Philadelphia; 10 Division of Gastroenterology, University of Pennsylvania, Philadelphia; 11 Department of Biostatistics and Epidemiology, University of Pennsylvania, Philadelphia; 12 Center for Innovations in Quality, Effectiveness, and Safety, Veterans Affairs Puget Sound Health Care System, Seattle, Washington; 13 Department of Internal Medicine, Division of Gastroenterology, University of Washington School of Medicine, Seattle; 14 Michigan Integrated Center for Health Analytics and Medical Prediction, Ann Arbor

## Abstract

**Importance:**

Inflammatory bowel disease (IBD) is commonly treated with corticosteroids and anti–tumor necrosis factor (TNF) drugs; however, medications have well-described adverse effects. Prior work suggests that anti-TNF therapy may reduce all-cause mortality compared with prolonged corticosteroid use among Medicare and Medicaid beneficiaries with IBD.

**Objective:**

To examine the association between use of anti-TNF or corticosteroids and all-cause mortality in a national cohort of veterans with IBD.

**Design, Setting, and Participants:**

This cohort study used a well-established Veteran’s Health Administration cohort of 2997 patients with IBD treated with prolonged corticosteroids (≥3000-mg prednisone equivalent and/or ≥600 mg of budesonide within a 12-month period) and/or new anti-TNF therapy from January 1, 2006, to October 1, 2015. Data were analyzed between July 1, 2019, and December 31, 2020.

**Exposures:**

Use of corticosteroids or anti-TNF.

**Main Outcomes and Measures:**

The primary end point was all-cause mortality as defined by the Veterans Health Administration vital status file. Marginal structural modeling was used to compare associations between anti-TNF therapy or corticosteroid use and all-cause mortality.

**Results:**

A total of 2997 patients (2725 men [90.9%]; mean [SD] age, 50.0 [17.4] years) were included in the final analysis, 1734 (57.9%) with Crohn disease (CD) and 1263 (42.1%) with ulcerative colitis (UC). All-cause mortality was 8.5% (n = 256) over a mean (SD) of 3.9 (2.3) years’ follow-up. At cohort entry, 1836 patients were new anti-TNF therapy users, and 1161 were prolonged corticosteroid users. Anti-TNF therapy use was associated with a lower likelihood of mortality for CD (odds ratio [OR], 0.54; 95% CI, 0.31-0.93) but not for UC (OR, 0.33; 95% CI, 0.10-1.10). In a sensitivity analysis adjusting prolonged corticosteroid users to include patients receiving corticosteroids within 90 to 270 days after initiation of anti-TNF therapy, the OR for UC was statistically significant, at 0.33 (95% CI, 0.13-0.84), and the OR for CD was 0.55 (95% CI, 0.33-0.92).

**Conclusions and Relevance:**

This study suggests that anti-TNF therapy may be associated with reduced mortality compared with long-term corticosteroid use among veterans with CD, and potentially among those with UC.

## Key Points

**Question:**

Is there an association between the use of anti–tumor necrosis factor (TNF) therapy and all-cause mortality in a national cohort of patients with inflammatory bowel disease (IBD)?

**Findings:**

In this cohort study of 2297 patients, all-cause mortality was 9% over a mean follow-up of of 3.9 years. Anti-TNF therapy was associated with a lower likelihood of mortality for Crohn disease but not ulcerative colitis.

**Meaning:**

This study suggests that anti-TNF therapy may be associated with reduced mortality compared with long-term corticosteroid use among veterans with Crohn disease.

## Teaser

This cohort study examines the association between use of anti–tumor necrosis factor (TNF) therapy or corticosteroids and all-cause mortality in a national cohort of veterans with inflammatory bowel disease (IBD).

## Introduction

Inflammatory bowel disease (IBD) is a chronic inflammatory condition associated with disability and reduced quality of life.^1^ Corticosteroids temporarily treat inflammation, but guidelines recommend against their long-term use because of their toxicity profile and ineffectiveness at maintaining clinical remission (the resolution of symptoms).^2,3^ Patients with moderate to severe IBD are often prescribed corticosteroid-sparing therapies, such as anti–tumor necrosis factor (TNF) agents, for the induction and maintenance of clinical remission. However, many patients do not respond to anti-TNF agents or develop recurrent inflammation despite them, prompting reinitiation of corticosteroids.^4,5^

Both corticosteroids and anti-TNF agents have adverse effects, including infections (both), osteoporosis (corticosteroids), and congestive heart failure (both).^6,7,8^ A study of Medicaid and Medicare beneficiaries found a decreased risk of death among patients with Crohn disease (CD) exposed to anti-TNF agents, compared with those taking corticosteroids for a prolonged time.^9^ Prior registry studies show similar findings for immunomodulators and the anti-TNF medication infliximab, compared with corticosteroids.^10,11^ Furthermore, recent data suggest that corticosteroid use is also associated with a 6-fold increased risk of severe coronavirus disease 2019 (COVID-19), wherease anti-TNF agent use is not.^12^ Despite this, longstanding corticosteroid use among patients with IBD remains common, particularly among the elderly and those with comorbid conditions.^13,14^

The Veterans Health Administration (VHA) is the largest integrated health system in the United States, serving 9 million veterans.^15^ Veterans with IBD frequently have risk factors predisposing them to both corticosteroid and anti-TNF agent adverse effects, including older age, tobacco use, and comorbidities, such as coronary artery disease, hypertension, hyperlipidemia, and diabetes.^7,16,17^ We aimed to examine the hypothesis that anti-TNF therapy is associated with reduced mortality relative to prolonged corticosteroid use in an established cohort of veterans with IBD.

## Methods

Veterans with IBD followed up within VHA were identified using *International Classification of Diseases, Ninth Revision* (*ICD-9*) codes for CD (555.x) and ulcerative colitis (UC) (556.x). Patients were included if they had 2 or more clinical encounters within the VHA associated with these codes between January 1, 2006, and October 1, 2015, with 1 or more encounter being an outpatient visit.^7^ This algorithm has demonstrated a positive predictive value of 0.84 for CD and 0.91 for UC.^18^ Veterans were classified as having undefined IBD if both CD and UC codes were present and were excluded from the final cohort. The Internal Review Board at the VA Ann Arbor Healthcare System Research Services approved this study for a waiver of informed consent for the following reasons: the study involved no more than minimal risk to the participants; the waiver will not adversely affect the rights and welfare of the participants; and the research could not practicably be conducted without the waiver and without access to or use of the PHI. Whenever appropriate, the participants (including their physicians, as applicable) were provided with additional pertinent information after participation. There was no contact with patients; only existing data were collected, and therefore no participant rights and/or welfare were affected. The research did not impact clinical care decisions or access to clinical care in any way. This report follows the Strengthening the Reporting of Observational Studies in Epidemiology (STROBE) reporting guideline.

### Inclusion Criteria

Our cohort included patients with incident IBD with prolonged corticosteroid use and/or new anti-TNF use (eTable 1 in the Supplement), stratified by IBD type. Prolonged corticosteroid use was defined as 3000 mg or more of prednisone or equivalent (eTable 2 in the Supplement) and/or 600 mg or more of budesonide, spread over 2 or more prescriptions within 12 months.^9^ These definitions were based on typical IBD tapering dosages.^9^ Topical, otic, ophthalmic, and inhaled formulations were excluded. New anti-TNF use was defined as 1 or more filled prescription for adalimumab, certolizumab, or infliximab during the study period, with no anti-TNF prescriptions filled in the preceding 12 months. We did not include golimumab because it was not in the VHA formulary during our study period. Follow-up began when a patient met criteria for either prolonged corticosteroid use or new anti-TNF agent use.

Patients who entered the cohort with prolonged corticosteroid use and subsequently began using a new anti-TNF agent contributed follow-up time to the corticosteroid group until the date of their first anti-TNF agent prescription fill, at which point they began contributing follow-up time to the anti-TNF agent group. Such patients continued to contribute time to the anti-TNF agent group thereafter, even if they subsequently discontinued the anti-TNF agent. Patients who entered the cohort with new anti-TNF use remained in this group for the entire duration of their follow-up, even if they subsequently met inclusion criteria for prolonged corticosteroid use. This approach allowed us to isolate the degree to which any anti-TNF agent exposure was associated with mortality, independent of duration of exposure or association with symptoms.

### Exclusion Criteria

We excluded patients with unverified dates of birth or who were 90 years of age or older because older age is associated mortality. We also excluded patients with IBD diagnosed before 2006, rheumatoid arthritis, psoriasis, psoriatic arthritis, ankylosing spondylitis, systematic lupus erythematosus, Paget disease, asthma, chronic obstructive pulmonary disease, HIV, multiple sclerosis, or a malignant neoplasm during the 12 months prior to cohort entry, given their association with mortality and/or competing indication for corticosteroid or anti-TNF therapy.^9^

### Outcome

The primary end point was all-cause mortality as defined by the VHA vital status file.^19^ We censored patients at 90 years of age; at diagnosis of HIV, rheumatoid arthritis, psoriatic arthritis, psoriasis, or ankylosing spondylitis; or at the end of the study period (October 1, 2015).

### Covariates

We evaluated 55 covariates, including demographic characteristics, year at study entry, and additional variables that might be associated with a clinician’s IBD treatment choice, risk of mortality, or treatment complications (eTable 3 in the Supplement).^9^ Race/ethnicity was classified based on veteran self-report as captured in VHA data. All nondemographic covariates were captured from administrative data, using *ICD-9* and *Current Procedural Terminology* (*CPT*) codes. We also included year of cohort entry, given evolving trends in use of anti-TNF therapy during the study period. Definitions for all covariates are outlined in eTable 3 in the Supplement.

### Statistical Analysis

Data were analyzed between July 1, 2019, and December 31, 2020. We used marginal structural modeling to estimate the association between anti-TNF agent use and mortality, stratified by IBD type. Marginal structural modeling uses weighted individual-level treatment effects prior to calculating population effects, allowing the model to correct for time-varying variables that are associated with future treatment and prior treatment, such as disease severity and disease-related and treatment-related complications.^9,20^ We derived stabilized weights using separate models for both inverse probability of treatment and inverse probability of remaining uncensored. Weights were estimated separately for patients with CD and those with UC. Time-varying covariates were updated every 28 days.

To construct a stabilized inverse probability of treatment weights, we first constructed propensity scores by estimating the probability of receiving a corticosteroid and anti-TNF agent at each point.^20^ Patients with treatment probabilities beyond the 2nd and 98th percentiles were excluded to prevent those with very low or high likelihood of receiving treatment from unduly influencing model weights. We calculated weighted standardized mean differences (SMDs) for all covariates, with an SMD less than 0.10 indicating a reasonable balance of covariates from baseline propensity models.^21^ The probability of censoring was estimated in a similar manner. Nine baseline covariates with an SMD of more than 0.10 were identified: comorbidity score, hypertension, coronary disease, hypercholesterolemia, anemia, colonoscopy, computed tomography scan, prior corticosteroid use, and other medication prescriptions (Table 1; eTable 4 in the Supplement). To reduce residual imbalance, we included covariates in our final model.^22^ Treatment and censoring weights were multiplied and accumulated at each follow-up period, with the resulting weight representing each patient’s treatment trajectory. We again truncated weights as already mentioned.

**Table 1.** Patient Characteristics Assessed During the 12 Months Prior to Enrollment

Characteristic	No. (%) of patients with Crohn disease (n = 1734 [57.9%])		SMD	No. (%) of patients with ulcerative colitis (n = 1263 [42.1%])		SMD
	New anti-TNF users (n = 875)	Prolonged CS users (n = 859)		New anti-TNF users (n = 286)	Prolonged CS users (n = 977)	
Age, y						
19-35	323 (36.9)	179 (20.8)	0.27	98 (34.3)	216 (22.1)	0.23
36-50	223 (25.5)	150 (17.5)		62 (21.7)	190 (19.4)	
51-65	202 (23.1)	280 (32.6)		63 (22.0)	296 (30.3)	
66-70	77 (8.8)	102 (11.9)		32 (11.2)	103 (10.5)	
71-79	25 (2.9)	60 (7.0)		17 (5.9)	67 (6.9)	
81-85	16 (1.8)	38 (4.4)		9 (3.1)	46 (4.7)	
>85	5 (0.6)	34 (4.0)		3 (1.0)	41 (4.2)	
Age, mean (SD), y	44.4 (16.0)	53.35 (17.2)		47.09 (17.42)	52.81 (17.5)	
White race	673 (76.9)	662 (77.1)	0.18	227 (79.4)	745 (76.3)	
Calendar year at entry						
2007	35 (4.0)	116 (13.5)	0.01	8 (2.8)	100 (10.2)	0.12
2008	57 (6.5)	123 (14.3)		8 (2.8)	106 (10.8)	
2009	80 (9.1)	100 (11.6)		15 (5.2)	113 (11.6)	
2010	84 (9.6)	110 (12.8)		23 (8.0)	138 (14.1)	
2011	112 (12.8)	93 (10.8)		29 (10.1)	120 (12.3)	
2012	135 (15.4)	108 (12.6)		35 (12.2)	121 (12.4)	
2013	152 (17.4)	111 (12.9)		70 (24.5)	130 (13.3)	
2014	220 (25.1)	98 (11.4)		98 (34.3)	149 (15.3)	
Male	765 (87.4)	779 (90.7)	0.10	262 (91.6)	919 (94.1)	
Charlson comorbidity count						
0	621 (71.0)	534 (62.2)	0.05	197 (68.9)	608 (62.2)	0.03
1	171 (19.5)	191 (22.2)		63 (22.0)	206 (21.1)	
2-3	70 (8.0)	110 (12.8)		22 (7.7)	126 (12.9)	
>3	13 (1.5)	24 (2.8)		4 (1.4)	37 (3.8)	
Medications used						
Azathioprine or mercaptopurine	283 (32.3)	326 (38.0)	0.16	100 (35.0)	336 (34.4)	0.10
Budesonide	46 (5.3)	442 (51.5)	0.18	13 (4.5)	139 (14.2)	0.19
Fibrate	20 (2.3)	34 (4.0)	0.05	2 (0.7)	19 (1.9)	0.08
Methotrexate	23 (2.6)	13 (1.5)	0.09	1 (0.3)	8 (0.8)	0.07
Metronidazole	176 (20.1)	187 (21.8)	0.08	43 (15.0)	233 (23.8)	0.03
Narcotic	348 (39.8)	364 (42.4)	0.08	81 (28.3)	375 (38.4)	0.02
Quinolone	139 (15.9)	168 (19.6)	0.08	31 (10.8)	188 (19.2)	0.13
Prednisone	239 (27.3)	401 (46.7)	0.28	120 (42.0)	792 (81.1)	0.14
No hospitalizations for IBD	803 (91.8)	759 (88.4)	0.05	270 (94.40)	828 (84.7)	0.04
≥1 Non-IBD hospitalization	63 (7.2)	87 (10.1)	0.09	20 (7.0)	96 (9.8)	0.03
First drug qualifying for enrollment						
Adalimumab	699 (79.9)	NA	NA	206 (72.0)	NA	NA
Prednisone	NA	343 (39.9)	NA	NA	825 (84.4)	NA
Infliximab	120 (13.7)	NA	NA	79 (27.6)	NA	NA
Budesonide	NA	516 (60.1)	NA	NA	152 (15.6)	NA
Certolizumab	56 (6.4)	NA	NA	1 (0.3)	NA	NA

Abbreviations: CS, corticosteroids; IBD, inflammatory bowel disease; NA, not applicable; SMD, standardized mean difference, after model weights applied; TNF, tumor necrosis factor.

For the primary analysis, estimated using a survey-weighted generalized linear model approach to account for clustering of observations within patients, inverse probability of treatment weights and design-based SEs were used to obtain 95% CIs. SEs reflect the possibility that individual patients could contribute to more than 1 exposure group. The odds ratios (ORs) from these models are approximate hazard ratios, derived using a discrete-time approach.

We performed 2 sensitivity analyses to evaluate the association of patients entering the cohort with prolonged corticosteroid use, then starting a new anti-TNF agent. The first sensitivity analysis excluded patients continuing to take corticosteroids 90 to 270 days after initiation of anti-TNF therapy. The second analysis reclassified all follow-up time for these patients as contributing to the prolonged corticosteroid group, instead of reassigning them to the anti-TNF agent group after they filled an anti-TNF agent prescription. In a third analysis, we accounted for duration of anti-TNF agent exposure by censoring all users on the date of their last anti-TNF prescription fill. In a final sensitivity analysis, we restricted our corticosteroid definition to exclude budesonide, given the differences in first-pass metabolism. We tested for differences in medication exposure (anti-TNF agent vs nonbudesonide corticosteroids) using 2-tailed *t* tests, with *P* < .05 considered statistically significant. Statistical analysis was performed in R, version 3.6.0 (R Project for Statistical Computing).

## Results

We identified 42 999 patients with a diagnosis of IBD during the study period, among whom 36 728 had available demographic data (eTable 4 in the Supplement). Most were White individuals (25 614 [69.7%]) and men (34 271 [93.3%]), with a mean (SD) age of 60.4 (15.1) years. We excluded 1468 patients for other immune-mediated diseases; 915 patients for presence of Paget disease, asthma, or chronic obstructive pulmonary disease; 32 patients with HIV or AIDS, multiple sclerosis, or malignant neoplasm; and 13 patients who were 90 years or older or without a verifiable age. We excluded 1326 patients with IBD diagnosed before the study period and 763 patients with undefined IBD. A total of 2997 patients initiated a corticosteroid or anti-TNF agent during the study period and were included in the final analysis: 1734 (57.9%) with CD and 1263 (42.1%) with UC (Table 1; Figure). In this cohort, 2307 (77.0%) were White individuals, 2725 (90.9%) were men, and the mean (SD) age was 50.0 (17.4) years. Overall, 1836 patients (61.3%) were new anti-TNF therapy users and 1161 (38.7%) were prolonged corticosteroid users. Among patients with UC, 977 (77.4%) were classified as prolonged corticosteroid users and 286 (22.6%) as new anti-TNF users; among those with CD, 859 (49.5%) were classified as prolonged corticosteroid users and 875 (50.5%) as new anti-TNF users.

**Figure.**
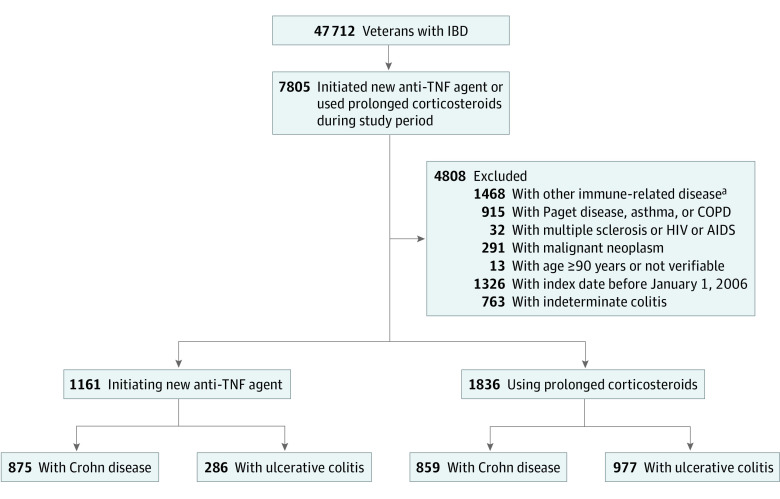
Study Population With Exclusions and Inclusions COPD indicates chronic obstructive pulmonary disease; IBD, inflammatory bowel disease; and TNF, tumor necrosis factor. ^a^Rheumatoid arthritis, psoriasis, psoriatic arthritis, ankylosing spondylitis, and lupus.

During the study period, 445 patients who were initially classified as prolonged corticosteroid users (251 patients with CD [56.4%] and 194 patients with UC [43.6%]) started a new anti-TNF agent. Of these 445 patients, 213 received additional corticosteroids within the first 6 months. The mean (SD) age for these patients was 46.4 (16.0) years, and 16 (3.6%) died during the study period. The mean (SD) follow-up time accrued between classification as a prolonged corticosteroid user and initiation of anti-TNF therapy was 2.8 (2.4) years. The highest frequency of new anti-TNF usen in this group occurred in 2014 (19.8% [88 of 445]), and the lowest frequency occurred in 2007 (2.2% [10 of 445]).

For both CD and UC, patients entering as prolonged corticosteroid users were older and had poorer health than new anti-TNF users, including higher number of comorbidities, more non–IBD-associated medications, and higher rates of non–IBD-related hospitalizations. They were more likely to use narcotics and to enroll earlier in the study period. Patients using anti-TNF agents had more IBD-related hospital days and electrolyte disorders and were more likely to undergo a colonoscopy. Patients with CD were younger and had fewer comorbidities than those with UC, used more narcotics, and enrolled earlier in the study period. Patients with CD had more serious infections than those with UC, but exposure to immunomodulators and non-IBD medication use overall were similar across groups.

Median daily doses of corticosteroids are reported in Table 2. Among patients entering with prolonged corticosteroid use, patients with CD received a higher mean (SD) dose of budesonide in the 12 months preceding study entry than those with UC (4.4 [4.3] mg/d vs 1.2 [3.0] mg/d) but a lower mean (SD) dose of prednisone or equivalent during this period (13.4 [17.9] mg/d vs 24.8 [17.4] mg/d). Similar trends were observed in the first year after study entry, although mean daily doses of both budesonide and prednisone decreased from the first 6 months of the study period to the subsequent 6 months. Patients entering the study as anti-TNF users were unlikely to use budesonide during the year prior to or after cohort enrollment. Patients using anti-TNF agents who used prednisone had lower mean (SD) daily doses than patients who entered with prolonged corticosteroid use (CD, 8.3 [22.4] mg/d; UC, 11.3 [16.6] mg/d) during the year prior to enrollment. Trends for the 1 to 6 months and 7 to 12 months after cohort entry were similar (Table 2). Differences in mean daily corticosteroid dosing were significantly higher in the prolonged corticosteroid group compared with the new anti-TNF group.

**Table 2.** Description of Corticosteroid Use Stratified by Treatment Group

Treatment group	Prolonged corticosteroid use		New anti-TNF agent use		*P* value
	Median (IQR)	Mean (SD)	Median (IQR)	Mean (SD)	
**Crohn disease**					
Budesonide, mg					
0-12 mo Before entry	5.3 (0-9.0)	4.4 (4.3)	0	0.5 (2.0)	<.001
1-6 mo After entry	6.0 (0-9.0)	4.8 (4.2)	0	0.3 (1.7)	<.001
7-12 mo After entry	0 (0-6.0)	2.6 (3.9)	0	0.4 (1.8)	<.001
Systemic Corticosteroid, mg prednisone equivalent					
0-12 mo Before entry	0 (0-24.5)	13.4 (17.9)	0 (0-9.5)	8.3 (22.4)	<.001
1-6 mo After entry	0 (0-22.3)	12.8 (18.6)	0	6.6 (45.8)	<.001
7-12 mo After entry	0	4.7 (12.2)	0	3.5 (14.2)	.06
**Ulcerative colitis**					
Budesonide, mg					
0-12 mo Before entry	0	1.2 (3.0)	0	0.3 (1.6)	<.001
1-6 mo After entry	0	1.3 (3.0)	0	0.3 (1.7)	<.001
7-12 mo After entry	0	0.6 (2.1)	0	0.4 (2.1)	.10
Systemic corticosteroid, mg prednisone equivalent					
0-12 mo Before entry	25.0 (14.3-35.0)	24.8 (17.4)	0 (0-21.5)	11.3 (16.7)	<.001
1-6 mo After entry	23.3 (13.0-35.9)	25.1 (18.8)	0 (0-19.3)	10.6 (19.0)	<.001
7-12 mo After entry	0 (0-15.4)	9.0 (15.1)	0	4.2 (12.2)	<.001

Abbreviations: IQR, interquartile range; TNF, tumor necrosis factor.

Overall mortality in the study population was 8.5% (n = 256). Similar rates were seen among patients with CD (8.5% [n = 147]) and patients with UC (8.6% [n = 109]). The mean (SD) follow-up time was 3.9 (2.3) years for patients with CD and patients with UC. Among those who died during follow-up, the mean (SD) age at death was 69.1 (14.0) years overall, 66.7 (15.0) years for those with CD, and 72.2 (11.7) years for those with UC. After controlling for covariates (eTable 3 in the Supplement), we found that the adjusted mortality OR [aOR] for initiating anti-TNF therapy compared with receiving corticosteroids was 0.54 (95% CI, 0.31-0.93) in the CD cohort and 0.33 (95% CI, 0.10-1.10) in the UC cohort. We performed 4 sensitivity analyses evaluating the association of persistent corticosteroid use after initiation of anti-TNF therapy. In the first, we excluded 152 patients (86 [56.6%] with CD and 66 [43.4%] with UC) who continued to use corticosteroids 90 to 270 days after initiation of anti-TNF therapy. This excluded 5.0% of patients with CD and 5.2% with UC. The aOR was 0.55 (95% CI, 0.33-0.92) for CD and 0.33 (95% CI, 0.13-0.84) for UC. In the second sensitivity analysis, we retained these 152 patients in the cohort but reclassified their entire follow-up time as contributing toward prolonged corticosteroid treatment, resulting in similar estimates (CD: aOR, 0.57 [95% CI, 0.34-0.96]; UC: aOR, 0.34 [95% CI, 0.13-0.87]). In a third analysis accounting for duration of anti-TNF agent exposure, initiation of anti-TNF therapy was associated with an aOR of 0.23 (95% CI, 0.06-0.88) for UC and an aOR of 0.52 (95% CI, 0.22-1.24) for CD. In a final sensitivity analysis in which the definition of corticosteroids excluded budesonide, the aOR for UC was 0.40 (95% CI, 0.12-1.27), and the aOR for CD was 0.22 (95% CI, 0.09-0.53).

## Discussion

The initiation of anti-TNF therapy was associated with a reduced likelihood of mortality compared with long-term corticosteroid use among patients with CD in a well-established longitudinal cohort of veterans with IBD. We saw a nonsignificant reduction in mortality among anti-TNF agent users with UC in our primary analysis, which became significant in sensitivity analyses accounting for persistent corticosteroid exposure after initiation of anti-TNF therapy.

Although corticosteroids and anti-TNF agents both have associated adverse effects, corticosteroids have a less favorable risk-benefit ratio for IBD.^6,9,10^ However, the protective role of anti-TNF agents against the harmful effects of corticosteroids, especially mortality, is less established among patients at the highest risk of medication adverse effects. The association of anti-TNF agents with mortality, compared with corticosteroids, is especially relevant in the care of patients who are elderly or have multiple morbidities, for whom concerns regarding the safety of anti-TNF agents arise, and clinicians are often more comfortable prescribing corticosteroids. Such dilemmas often arise when treating veterans with IBD, making this an appropriate population in which to further study the risk of initiating anti-TNF agents compared with maintaining corticosteroids.^23^ Compared with new anti-TNF users, prolonged corticosteroid users were older, had more comorbidities, and had higher overall health care use as measured by medication use and hospitalizations. These trends suggest that concerns about adverse events among patients with more severe illness may be associated with anti-TNF therapy.

Our work shows trends in the veteran population that are similar to, but more pronounced than, those that Lewis et al^9^ observed in Medicare and Medicaid beneficiaries (CD: OR, 0.87 [95% CI, 0.63-0.91]; UC: OR, 0.87 [95% CI, 0.63-1.22]). There are key differences between the populations evaluated in these studies, which may have contributed to the differences in results. First, our study period for both UC and CD encompassed 2006 to 2014, whereas Lewis et al^9^ incorporated the period from 2001 to 2005 (Medicaid beneficiaries with CD) and from 2006 to 2013 (Medicare beneficiaries with UC and CD). The last 2 decades have shown considerable changes in IBD care, with the increase in the use of corticosteroid-sparing therapy associated with improved disease-related morbidity and shorter duration of cumulative corticosteroid exposure.^24,25^ This conjecture is further supported by the appearance of a protective association of anti-TNF therapy with rates of hip fracture and cardiovascular disease in the earlier-enrolling cohort of patients with CD but not the later-enrolling cohort of patients with UC in the study by Lewis et al.^9^ In addition, 58% of veterans initiating anti-TNF agents in our cohort had no captured corticosteroid exposure. This finding is supported by extremely low rates of corticosteroid use, relative to civilian cohorts, among veterans initiating third-line treatments, such as ustekinumab (13% vs 35%-45%).^26,27^ This seeming paradox may be due to corticosteroid prescribing by comanaging civilian gastroenterologists or primary care physicians not captured in VHA claims. Fortunately, any misclassification would bias the findings toward the null.

### Strengths and Limitations

Our study has several unique strengths. The study period focuses on the biologic era and thus reflects current patterns of IBD management, with less likelihood of confounding due to prolonged corticosteroid use preceding enrollment. We used a well-established cohort of patients with IBD and were able to follow up with patients in our cohort for a mean (SD) of 3.9 (2.3) years after IBD diagnosis, allowing excellent capture of mortality rates, comorbidities, and medication use.^7,28,29^ Our use of a marginal structural model allowed us to adjust for the time-varying association of treatment exposure with our outcome, which is not possible using models such as a Cox proportional hazards regression. Finally, we demonstrated consistent results across different definitions of prolonged corticosteroid use in our 2 sensitivity analyses.

This study has several limitations. As with any administrative data set, *ICD-9* and *CPT* codes were used to identify patients with IBD, although the algorithm used has been well validated in this cohort.^18^ Residual confounding by coding errors, disease activity, reason for medication discontinuation, patient and clinician preferences, and system-level issues, such as specialty care access and continuity of care, is possible. Because anti-TNF treatment was initiated, on average, later during the study period, there is also possible unmeasured confounding due to differences in follow-up time between the corticosteroid and anti-TNF treatment groups. Our study population was limited to veterans receiving care within the VHA, and our results may thus not be generalizable to all patients with IBD within the community. We were limited by sample size, which may have contributed to the lack of a significant mortality reduction seen in the group of patients with UC and may have limited our ability to assess factors such as exposure period, dose, and subtype of anti-TNF agent. We also did not include data on smoking owing to unreliable capture within administrative claims.

## Conclusions

We found a robust association between anti-TNF use and reduced mortality among patients with CD in a well-established longitudinal cohort of veterans with IBD, with a nonsignificant mortality reduction among patients with UC. This finding supports prior high-quality studies of Medicaid and Medicare beneficiaries. Given the observation that older patients with more comorbidities do not seem to receive anti-TNF agents to the same degree as their younger, healthier counterparts, there is an urgent need to recognize the benefit associated with anti-TNF therapy vs corticosteroid use in such populations. A multicenter prospective cohort study is warranted to evaluate how factors such as IBD severity, year of diagnosis, and corticosteroid exposure after initiation of anti-TNF agent are associated with risk of mortality and treatment adverse effects.

## Notes

Supplement. **eTable 1.** Study Medications **eTable 2.** Prednisone-Equivalent Dosages of Systemic Corticosteroid Medications **eTable 3.** Definitions of Covariates Used **eTable 4.** Population Characteristics at Cohort Entry for All Included Covariates
