# Supplementary material for: Association of Anti–Tumor Necrosis Factor Therapy With Mortality Among Veterans With Inflammatory Bowel Disease
Source: JAMA Netw Open. 2021 Mar 1;4(3):e210313. doi: 10.1001/jamanetworkopen.2021.0313 (PMC7921894; doi:10.1001/jamanetworkopen.2021.0313)
Supplement: Supplement. — eTable 1. Study Medications eTable 2. Prednisone-Equivalent Dosages of Systemic Corticosteroid Medications eTable 3. Definitions of Covariates Used eTable 4. Population Characteristics at Cohort Entry for All Included Covariates [file jamanetwopen-e210313-s001.pdf]

Supplementary Online Content

Cohen-Mekelburg S, Wallace BI, Van T, et al. Association of anti–tumor necrosis factor therapy with mortality among veterans with inflammatory bowel disease. *JAMA Netw Open.* 2021;4(3):e210313. doi:10.1001/jamanetworkopen.2021.0313

eTable 1. Study Medications
eTable 2. Prednisone-Equivalent Dosages of Systemic Corticosteroid Medications
eTable 3. Definitions of Covariates Used
eTable 4. Population Characteristics at Cohort Entry for all Included Covariates

This supplementary material has been provided by the authors to give readers additional information about their work.

eTable 1. Study Medications

Corticosteroid medications	Dexamethasone, betamethasone, prednisone, prednisolone, methylprednisolone, cortisone, hydrocortisone, triamcinolone
Anti-TNF medications	infliximab, adalimumab, certolizumab pegol

TNF = tumor necrosis factor

© 2021 Cohen-Mekelburg S et al. *JAMA Network Open.*

eTable 2. Prednisone-Equivalent Dosages of Systemic Corticosteroid Medications

Dose	Glucocorticoid Name
0.6 mg	Betamethasone
0.75 mg	Dexamethasone
4 mg	Methylprednisolone
4 mg	Triamcinolone
5 mg	Prednisone
5 mg	Prednisolone
20 mg	Hydrocortisone
25 mg	Cortisone

© 2021 Cohen-Mekelburg S et al. *JAMA Network Open.*

eTable 3. Definitions of Covariates Used

Measurement indicates covariates also coded as time-varying (every 6 months).

Covariates	Definition
Carotid artery disease	**ICD9 code 433.1**
Carotid artery disease	ICD9 procedure 38.12, 00.63
Hypercholesterolemia*^	ICD9 code 272.0, 272.4, 272.2
Osteoporosis*^	ICD9 code 733
Coronary artery disease	410-412, 414.0-414.9 , 414.01
Parkinsons disease	332
History of stroke^	430.x, 431.x, 433.x1, 434.x1, 435.x, 436,
Problems with balance	781.2
Falls in prior 365 days	E880-E888
DEXA scan in prior 365 days*^	CPT codes (pre-2006) 76075 and (post-2006) 77080
Serious infection w/in 183 days prior to index^	The primary hospital discharge diagnosis must be for one of the below<br>• Meningitis – 320.x, 049.x<br>• Encephalitis – 323.x, 054.3<br>• Cellulitis – 681.x, 682.x<br>• Endocarditis – 421.x<br>• Pneumonia – 481.x, 482.x<br>• Pyelonephritis – 590.x<br>• Septic arthritis – 711.0x excluding 711.08<br>• Osteomyelitis – 730.0x, 730.1x, 730.2x<br>• Bacteremia – 038.x, 790.7
Opportunistic infection w/in 183 days prior to index	The primary hospital discharge diagnosis must be for infection with one of the following organisms, +/- prescription requirements as below<br>• Aspergillus -- 117.3 or 484.6 + prescription for posaconazole, itraconazole, or voriconazole within 90 days of the diagnosis code<br>• Blastomyces (116.0), Coccidiodes (114.x), Cryptococcus (117.5 or 321.0), Histoplasma (115.x): also require prescription for fluconazole, itraconazole, or voriconazole within 90 days of the diagnosis code<br>• Pneumocystis (136.3)<br>• Actinomyces (039.x)<br>• Legionella (482.84)<br>• Listeria (027.0)<br>• Nocardia (defined as actinomyces)<br>• Salmonella (003.x)<br>• Tuberculosis: 010.x – 018.x + prescription of pyrazinamide within 90 days of diagnosis code<br>• non-tuberculous mycobacteria: 031.x<br>• Toxoplasma (130.x)
Pyoderma Gangrenosum w/in 183 days prior to index	ICD-9 686.01
Stool culture w/in 183 days prior to index	CPT 87045
Diabetes	250.x, 357.2, 362.0, 362.0[1-7], 366.41, 962.3, E932.3 VA:
Hypertension	401.0-401.9
Coronary artery disease^	414.01
Congestive heart failure^	428.0 as primary dx for hospitalization,
Blood transfusion w/in 183 days prior to index	36430, 36440, 36450, 36444

© 2021 Cohen-Mekelburg S et al. *JAMA Network Open.*

Intravenous iron w/in 183 days prior to index^	J1439, J1756, J2916
Parenteral nutrition w/in 183 days prior to index^	S9364', 'S9365', 'S9366', 'S9367', 'S9368', 'B4164', 'B4168', 'B4172', 'B4176', 'B4178', 'B4180', 'B4185', 'B4189', 'B4193', 'B4197', 'B4199', 'B4220', 'B4222', 'B4224', 'B5000', 'B5100', 'B5200'
	ICD 9: Parenteral infusion of concentrated nutritional substances 99.15
Anemia from iron deficiency or NOS w/in 183 days prior to index*^	280.9
Bowel Resection	17.31, 17.34, 45.61, 45.62, 45.71, 45.74, 46.02, 46.04, 48.41, 48.42, 48.43, 48.49, 48.5, 48.51, 48.52, 48.59, 48.62-69
Ostomy	46.1x, 46.2x, 46.3x
Proctectomy Ileostomy (inpatient)	'4840','4842','4843','4850','4851','4852','4849','4859'
Proctectomy Ileostomy (outpatient)	'44155','44156','45121','44211'
Fistula w/in 365 & 56 days prior to index*^ "procedures for fistula drainage or seton placement in the prior 365 days "	ICD 9 dx: intestinal fistula 569.81, anal fistula 565.1; '5374', CPT: 46280, 46285
Malnutrition	263.0, 263.1, 263.8, 263.9
Electrolyte abnormality	276.1, 276.8
Small bowel follow-through exam w/in 365 & 56 days prior to index*^	CPT codes: 74250, 74245
Other nutritional anemia (e.g. B12) w/in 183 days prior to index^	281.9
Colonoscopy or sigmoidoscopy w/in 365 & 56 days prior to index*^	CPT codes: '44388', '44389', '44390', '44391', '44392', '44393', '44394', '45378', '45379', '45380', '45381', '45382', '45383', '45384', '45385', '45386', 'G0105', 'G0121', sigmoidoscopy: 45330, 45331, 45332, 45334
CT or MRI scan of abdomen/pelvis w/in 365 & 56 days prior to index*^	74176, 74177, 74178
C difficile w/in 183 & 56 days prior to index^	ICD9 code 008.45
Quinolone Rx w/in 183 days prior to index*^	
Metronidazole Rx w/in 183 days prior to index*^	
Other Antibiotic Rx w/in 183 days prior to index*^	
Herpes Antiviral Rx w/in 183 days prior to index^	acyclovir, valacyclovir, famciclovir
Antifungal Rx w/in 183 days prior to index^	VA drug class AM700
Mesalamine Rx w/in 183 days prior to index*^	
AZA/6MP Rx w/in 183 days prior to index*^	
Methotrexate Rx w/in 183 days prior to index^	
Narcotics Rx (excluding Belladonna/Opium) w/in 183 days prior to index*^	Dihydrocodeine<br>Fentanyl<br>Hydrocodone<br>Hydromorphone<br>Meperidine<br>Morphine<br>Oxycodone<br>Oxymorphone<br>Pentazocine<br>Propoxyphene<br>Tapentadol<br>Tramadol

© 2021 Cohen-Mekelburg S et al. *JAMA Network Open.*

PPI Rx w/in 183 days prior to index*^	esomeprazole, pantoprazole, lansoprazole, rabeprazole, omeprazole, dexlansoprazole, prilosec, prevacid, nexium, kapidex, zegerid, dexilant, protonix, aciphex
Benzodiazepine Rx w/in 183 days prior to index*^	
Bisphosphonate Rx w/in 183 days prior to index*^	ZOLEDRONIC ACID, etidronate, alendronate, risedronate, tiludronate
High dose Vitamin D Rx w/in 183 days prior to index	VA drug class VT500
Statins Rx w/in 183 days prior to index*^	atorvastatin, fluvastatin, lovastatin, pitavastatin, pravastatin, rosuvastatin, simvastatin, lipitor, zocor, mevacor
Fibrates Rx w/in 183 days prior to index^	fenofibrate, gemfibrozil
Prednisone use in 183 days prior to index*	
Budesonide use in 183 days prior to index*	
Number of non-IBD medication classes taking during 183 days prior to index*^	(excl IM600, GA400, MS190)
Hypovolemia or dehydration	276.52, 276.51

© 2021 Cohen-Mekelburg S et al. *JAMA Network Open.*

eTable 4. Population Characteristics at Cohort Entry for all Included Covariates
All covariates are assessed over the 12 months prior to enrollment. Standardized mean differences between new anti-TNF and prolonged corticosteroid (CS) users before (raw) and after (adjusted) baseline propensity weighting (weighted).

	Crohn's Disease				Ulcerative Colitis			
	TNF	CS	SMD		TNF	CS	SMD	
			Raw	Weighted			Raw	Weighted
**Age (%)**			0.5539	0.2745			0.3766	0.2335
**[18,35)**	323 (36.9)	179 (20.8)			98 (34.3)	216 (22.1)		
**[35,50)**	223 (25.5)	150 (17.5)			62 (21.7)	190 (19.4)		
**[50,65)**	202 (23.1)	280 (32.6)			63 (22.0)	296 (30.3)		
**[65,70)**	77 (8.8)	102 (11.9)			32 (11.2)	103 (10.5)		
**[70,75)**	25 (2.9)	60 (7.0)			17 (5.9)	67 (6.9)		
**[75,80)**	16 (1.8)	38 (4.4)			9 (3.1)	46 (4.7)		
**[80,85)**	5 (0.6)	34 (4.0)			3 (1.0)	41 (4.2)		
**[85,110)**	4 (0.5)	16 (1.9)			2 (0.7)	18 (1.8)		
**Race (%)**			0.1780	0.1801			0.1019	0.0749
**Black**	115 (13.1)	84 (9.8)			20 (7.0)	95 (9.7)		
**Other**	26 (3.0)	18 (2.1)			9 (3.1)	33 (3.4)		
**Unknown**	61 (7.0)	95 (11.1)			30 (10.5)	104 (10.6)		
**White**	673 (76.9)	662 (77.1)			227 (79.4)	745 (76.3)		
**Calendar year at index date (%)**			0.5546	0.0069			0.7245	0.1247
**2007**	35 (4.0)	116 (13.5)			8 (2.8)	100 (10.2)		
**2008**	57 (6.5)	123 (14.3)			8 (2.8)	106 (10.8)		
**2009**	80 (9.1)	100 (11.6)			15 (5.2)	113 (11.6)		
**2010**	84 (9.6)	110 (12.8)			23 (8.0)	138 (14.1)		
**2011**	112 (12.8)	93 (10.8)			29 (10.1)	120 (12.3)		
**2012**	135 (15.4)	108 (12.6)			35 (12.2)	121 (12.4)		
**2013**	152 (17.4)	111 (12.9)			70 (24.5)	130 (13.3)		
**2014**	220 (25.1)	98 (11.4)			98 (34.3)	149 (15.3)		
**Male (%)**	765 (87.4)	779 (90.7)	0.1045	0.1019	262 (91.6)	919 (94.1)	0.0953	0.0348
**Anemia by iron deficiency = yes (%)**	65 (7.4)	54 (6.3)	0.0452	0.1071	20 (7.0)	57 (5.8)	0.0473	0.0274
**Problems with balance = yes (%)**	1 (0.1)	5 (0.6)	0.0795	0.0035	2 (0.7)	7 (0.7)	0.002	0.0100
**Blood transfusion = yes (%)**	3 (0.3)	3 (0.3)	0.0011	0.0086	2 (0.7)	11 (1.1)	0.0449	0.0502
**Congestive heart failure = yes (%)**	5 (0.6)	16 (1.9)	0.1047	0.0591	1 (0.3)	22 (2.3)	0.0432	0.0953
**Coronary artery disease = yes (%)**	43 (4.9)	73 (8.5)	0.1437	0.1006	20 (7.0)	97 (9.9)	0.1056	0.1076
**Colonoscopy or sigmoidoscopy (%)**			0.1735	0.1966			0.204	0.2704
**none**	564 (64.5)	558 (65.0)			169 (59.1)	530 (54.2)		

© 2021 Cohen-Mekelburg S et al. *JAMA Network Open.*

**w/in 56 days prior**	123 (14.1)	78 (9.1)			52 (18.2)	140 (14.3)		
**w/in 57-365 days prior**	188 (21.5)	223 (26.0)			65 (22.7)	307 (31.4)		
**C. difficile (%)**			0.0406	0.0865			0.085	0.0729
**none**	858 (98.1)	844 (98.3)			276 (96.5)	928 (95.0)		
**w/in 56 days prior**	8 (0.9)	5 (0.6)			4 (1.4)	15 (1.5)		
**w/in 57-365 days prior**	9 (1.0)	10 (1.2)			6 (2.1)	34 (3.5)		
**CT scan or MRI (%)**			0.1840	0.0880			0.0968	0.1255
**none**	692 (79.1)	733 (85.3)			264 (92.3)	880 (90.1)		
**w/in 56 days prior**	97 (11.1)	54 (6.3)			10 (3.5)	35 (3.6)		
**w/in 57-365 days prior**	86 (9.8)	72 (8.4)			12 (4.2)	62 (6.3)		
**Diabetes = yes (%)**	80 (9.1)	101 (11.8)	0.0856	0.0210	31 (10.8)	137 (14.0)	0.0966	0.0467
**DEXA scan = yes (%)**	56 (6.4)	66 (7.7)	0.0502	0.0709	12 (4.2)	64 (6.6)	0.1046	0.0914
**Dehydration = yes (%)**	19 (2.2)	30 (3.5)	0.0797	0.0477	16 (5.6)	58 (5.9)	0.0147	0.0931
**Falls = yes (%)**	16 (1.8)	29 (3.4)	0.0973	0.0450	7 (2.4)	25 (2.6)	0.0071	0.1079
**Fistula (%)**			0.2314	0.0605			0.0952	<0.0001
**none**	804 (91.9)	833 (97.0)			285 (99.7)	976 (99.9)		
**w/in 56 days prior**	36 (4.1)	9 (1.0)			0 (0.0)	1 (0.1)		
**w/in 57-365 days prior**	35 (4.0)	17 (2.0)			1 (0.3)	0 (0.0)		
**Hypercholesterolemia = yes (%)**	140 (16.0)	184 (21.4)	0.1393	0.1479	63 (22.0)	283 (29.0)	0.1597	0.049
**Hypertension = yes (%)**	175 (20.0)	265 (30.8)	0.2511	0.1439	75 (26.2)	322 (33.0)	0.1479	0.0306
**IV iron = yes (%)**	6 (0.7)	5 (0.6)	0.0131	0.0003	4 (1.4)	2 (0.2)	0.1342	0.0069
**Small bowel follow through (%)**			0.0478	<0.0001			<0.0001	<0.0001
**none**	801 (91.5)	744 (86.6)			285 (99.7)	957 (98.0)		
**w/in 56 days prior**	19 (2.2)	31 (3.6)			0 (0.0)	3 (0.3)		
**w/in 57-365 days prior**	55 (6.3)	84 (9.8)			1 (0.3)	17 (1.7)		
**Stool culture = yes (%)**	9 (1.0)	27 (3.1)	0.1484	0.0805	13 (4.5)	65 (6.7)	0.0918	0.0198
**Osteoporosis = yes (%)**	0 (0.0)	0 (0.0)	-	-	0 (0.0)	0 (0.0)	-	-
**Opportunistic infection = yes (%)**	0 (0.0)	0 (0.0)	-	-	2 (0.7)	3 (0.3)	-	-
**Parenteral nutrition = yes (%)**	1 (0.1)	1 (0.1)	0.0006	0.0021	0 (0.0)	0 (0.0)	-	-
**Serious infection = yes (%)**	38 (4.3)	27 (3.1)	0.0632	0.0041	8 (2.8)	33 (3.4)	0.0336	0.0251
**Bowel resection = yes (%)**	6 (0.7)	2 (0.2)	0.0670	0.0219	0 (0.0)	1 (0.1)	0.0453	<0.0001
**Nutritional anemia = yes (%)**	3 (0.3)	3 (0.3)	0.0011	0.1081	1 (0.3)	4 (0.4)	0.0097	0.0446
**Electrolyte disorders = yes (%)**	19 (2.2)	39 (4.5)	0.1318	0.0334	16 (5.6)	52 (5.3)	0.012	0.0334
**Benzodiazepine Rx = yes (%)**	105 (12.0)	127 (14.8)	0.0818	0.1791	29 (10.1)	145 (14.8)	0.1426	0.1650
**Bisphosphonate Rx = yes (%)**	14 (1.6)	37 (4.3)	0.1604	0.0495	1 (0.3)	27 (2.8)	0.1959	0.1446
**Statin = yes (%)**	95 (10.9)	149 (17.3)	0.1873	0.0786	48 (16.8)	261 (26.7)	0.2425	0.0548
**High dose vitamin D Rx = yes (%)**	110 (12.6)	96 (11.2)	0.0432	0.192	25 (8.7)	97 (9.9)	0.0408	0.0816
**Weight loss or malnutrition = yes (%)**	11 (1.3)	11 (1.3)	0.0021	0.1429	5 (1.7)	7 (0.7)	0.0936	0.0356
**Antibiotic Rx = yes (%)**	102 (11.7)	91 (10.6)	0.0338	0.1136	15 (5.2)	116 (11.9)	0.2386	0.0331
**Herpes antiviral Rx = yes (%)**	18 (2.1)	15 (1.7)	0.0228	0.0895	6 (2.1)	25 (2.6)	0.0306	0.0076
**Comorbidity score (%)**			0.1775	0.0547			0.1765	0.1126

© 2021 Cohen-Mekelburg S et al. *JAMA Network Open.*

**0**	621 (71.0)	534 (62.2)			197 (68.9)	608 (62.2)		
**1**	171 (19.5)	191 (22.2)			63 (22.0)	206 (21.1)		
**2-3**	70 (8.0)	110 (12.8)			22 (7.7)	126 (12.9)		
**4+**	13 (1.5)	24 (2.8)			4 (1.4)	37 (3.8)		
**AZA/ 6MP Rx = yes (%)**	283 (32.3)	326 (38.0)	0.1177	0.1613	100 (35.0)	336 (34.4)	0.0121	0.1082
**Budesonide use = yes (%)**	46 (5.3)	442 (51.5)	1.1936	0.1841	13 (4.5)	139 (14.2)	0.3366	0.1944
**Fibrates Rx = yes (%)**	20 (2.3)	34 (4.0)	0.0963	0.0563	2 (0.7)	19 (1.9)	0.1092	0.0876
**Methotrexate Rx = yes (%)**	23 (2.6)	13 (1.5)	0.0784	0.0947	1 (0.3)	8 (0.8)	0.0616	0.0783
**Metronidazole Rx = yes (%)**	176 (20.1)	187 (21.8)	0.0407	0.0801	43 (15.0)	233 (23.8)	0.2241	0.0309
**Narcotics Rx = yes (%)**	348 (39.8)	364 (42.4)	0.0529	0.0864	81 (28.3)	375 (38.4)	0.2146	0.0295
**Quinolone Rx = yes (%)**	139 (15.9)	168 (19.6)	0.0963	0.0826	31 (10.8)	188 (19.2)	0.2367	0.1394
**Pyoderma Gangrenosum = yes (%)**	3 (0.3)	2 (0.2)	0.0205	0.0032	0 (0.0)	3 (0.3)	0.0785	<0.0001
**Prednisone use = yes (%)**	239 (27.3)	401 (46.7)	0.4095	0.2805	120 (42.0)	792 (81.1)	0.8777	0.1407
**PPI Rx = yes (%)**	310 (35.4)	382 (44.5)	0.1854	0.1621	95 (33.2)	407 (41.7)	0.1751	0.1042
**Number of non-IBD med classes during 183 days prior (%)**			0.3522	0.2117			0.5069	0.1641
**0-3**	306 (35.0)	175 (20.4)			108 (37.8)	172 (17.6)		
**4-6**	219 (25.0)	218 (25.4)			72 (25.2)	242 (24.8)		
**7-10**	168 (19.2)	219 (25.5)			52 (18.2)	245 (25.1)		
**10+**	182 (20.8)	247 (28.8)			54 (18.9)	318 (32.5)		
**Number of IBD hospitalization days = none (%)**	803 (91.8)	759 (88.4)	0.1143	0.0584	270 (94.4)	828 (84.7)	0.3200	0.0469
**Number of non IBD hospitalizations = 1+ (%)**	63 (7.2)	87 (10.1)	0.1042	0.0913	20 (7.0)	96 (9.8)	0.1022	0.0397
**First drug that qualified patient into steroids or anti-TNF cohorts (%)**			-	-			-	-
**Adalimumab**	699 (79.9)	0 (0.0)			206 (72.0)	0 (0.0)		
**Prednisone**	0 (0.0)	343 (39.9)			0 (0.0)	825 (84.4)		
**Infliximab**	120 (13.7)	0 (0.0)			79 (27.6)	0 (0.0)		
**Budesonide**	0 (0.0)	516 (60.1)			0 (0.0)	152 (15.6)		
**Certolizumab Pegol**	56 (6.4)	0 (0.0)			1 (0.3)	0 (0.0)		

TNF = new anti-TNF users; CS = prolonged CS users

© 2021 Cohen-Mekelburg S et al. *JAMA Network Open.*
